# Long‐lasting changes in circulating dendritic cell and monocyte subsets, and altered expression of EMR2, CD97 and EMR3 on these cells in the posttraumatic course

**DOI:** 10.1002/cti2.70040

**Published:** 2025-06-17

**Authors:** Leyu Zheng, Carolin Fuchs, Christian Kleber, Georg Osterhoff, Gabriela Aust

**Affiliations:** ^1^ Research Laboratories and Department of Orthopaedics, Trauma and Plastic Surgery Leipzig University and University Hospital Leipzig Leipzig Germany; ^2^ Research Laboratories and Department of Visceral, Transplantation, Vascular and Thoracic Surgery Leipzig University and University Hospital Leipzig Leipzig Germany

**Keywords:** CD97, dendritic cells, EMR2, EMR3, monocyte subset, traumatic injury

## Abstract

**Objectives:**

Traumatic injury triggers the rapid release of damage‐associated patterns (DAMPs). Dendritic cells (DCs) and monocytes play key roles in sensing, processing, and presenting DAMPs to naïve T cells. These heterogeneous immune cells express the adhesion GPCR EMR2/*ADGRE2*, which is likely regulated by DAMPs.

**Methods:**

We analysed the various blood DC and monocyte subsets in trauma patients and uninjured volunteers using flow cytometry. EMR2 and its closest relatives, CD97/*ADGRE5* and EMR3/*ADGRE3*, were quantified on these subsets to gain insights into their (patho)physiological regulation.

**Results:**

Following trauma, conventional and plasmocytoid DCs nearly disappeared from the circulation, which is inversely correlated with injury severity and adverse clinical parameters 120–240 h post injury. Alterations in EMR2 and CD97 on DCs were relatively minor. Classical monocytes increased, while non‐classical monocytes showed a sustained decline in both absolute number and percentage, in a manner dependent on injury severity after trauma. EMR2 expression increased across all monocyte subsets, whereas CD97 showed little change. EMR3 expression decreased and remained low in classical monocytes, while it markedly increased in non‐classical monocytes. These temporal patterns in adhesion GPRC expression were largely independent of injury severity and were observed in all injured patients.

**Conclusion:**

Circulating DC and monocyte subsets underwent significant compositional changes after trauma, often correlating with injury severity and other clinical parameters. Despite structural similarities, EMR2, CD97, and EMR3 showed distinct regulatory patterns on monocyte subsets, suggesting different functional roles in the immune response to injury.

## Graphical abstract

We monitored various dendritic cell (DC) and monocyte subsets in traumatised patients for up to 240 h after injury, and compared them with uninjured volunteers who underwent the same blood sampling schedule. Simultaneously, EMR2, CD97, and EMR3 expression on these subsets was quantified by flow cytometry to gain insight into their (patho)physiological regulation and clinical relevance.

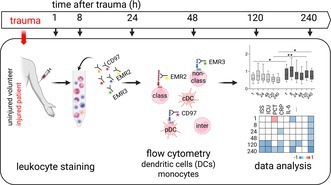

## Introduction

Trauma rapidly activates the innate immune response, facilitating the clearance of damaged tissues and cells along with their released contents, collectively known as damage‐associated molecular patterns (DAMPs). Immune cells are recruited to the site of injury, initiating sterile inflammation that ultimately leads to DAMP clearance, tissue repair and regeneration.

Dendritic cells (DCs) and monocytes play key roles in sensing and processing DAMPs. Both cell types present epitopes derived from internalised DAMPs in complex with MHC class II molecules to naïve T cells, thereby linking the innate and adaptive immune responses. DCs and monocytes are highly heterogeneous, comprising multiple subsets with distinct functions. Circulating DCs include CD11c^+^ conventional DCs (cDCs) and CD123^+^ plasmacytoid DCs (pDCs), which differ in their expression of various sensors, signalling pathways, and effectors, contributing to distinct immune responses.^1^, ^2^, ^3^ Notably, circulating DCs have not yet been systematically tracked following traumatic injury, likely because of their low abundance in peripheral blood.

Circulating monocytes are classified based on CD14 and CD16 expression into three subtypes: classical (CD14^hi^ CD16^−^), intermediate (CD14^hi^ CD16^+^), and non‐classical (CD14^lo^ CD16^+^) monocytes. CD14^hi^ monocytes exhibit a strong capacity for phagocytosis and endothelial transmigration, enabling their recruitment to injured tissues. In contrast, CD16^+^ monocytes patrol the vessel walls and contribute to endothelial maintenance. Importantly, high‐dimensional technologies have identified additional subsets of circulating DCs, monocytes and their progenitors, refining and expanding their classification.^4^, ^5^

Among the hundreds of GPCRs, adhesion GPCRs exhibit unique structural features and functions.^6^, ^7^ Their large N‐terminal extracellular domain (ECD) contains tandemly arranged adhesive folds, facilitating multiple cellular interactions, and the GPCR autoproteolysis domain, which permits self‐cleavage of the nascent receptor. EMR2, encoded by *ADGRE2*, as well as EMR3/*ADGRE3* and CD97/*ADGRE5*, contain multiple EGF‐like folds in their extracellular part. They are predominantly, or in the case of CD97, most highly expressed in circulating innate immune cells and originated through gene duplications and conversions in vertebrates.^8^ EMR2 is a structural chimera, with an extracellular domain that closely resembles CD97, while its seven‐transmembrane (7TM) region shares significant homology with EMR3.^8^

Our recent findings in injured patients indicate a marked upregulation of EMR2 on circulating neutrophils during the post‐traumatic course.^9^ This increase, peaking at 48 h after trauma, was observed across all patients with moderate to very severe injuries. Clinical and *in vitro* data suggest that DAMPs are the drivers of neutrophilic EMR2 upregulation.^9^ Notably, primary monocytes likely bind necrotic‐like cells *in vitro* via EMR2 in association with FHR1, a complement factor H‐related protein.^10^ EMR2 activation, either through interaction with FHR1 or through antibody‐mediated ligation, triggers NLRP3 inflammasome formation,^10^, ^11^ a key component of the innate immune response.

In contrast to EMR2, the (patho)physiological regulation and clinical significance of CD97 and EMR3 in circulating innate immune cells remain unclear or largely unknown. CD97 is highly expressed in circulating myeloid cells but has been infrequently studied in relevant clinical settings.^12^, ^13^ EMR3, which is restricted to blood monocytes and granulocytes in healthy individuals, is among the least explored adhesion GPCRs.^14^, ^15^

In this study, we tracked various DC and monocyte subsets in trauma patients for up to 240 h post injury, comparing them with uninjured volunteers who underwent the same blood sampling schedule. Simultaneously, we quantified EMR2, EMR3 and CD97 expression on these subsets to gain insights into their (patho)physiological regulation and clinical relevance.

## Results

### Posttraumatic injury‐dependent phenotypic changes in circulating monocyte subsets

**Figure 1:**
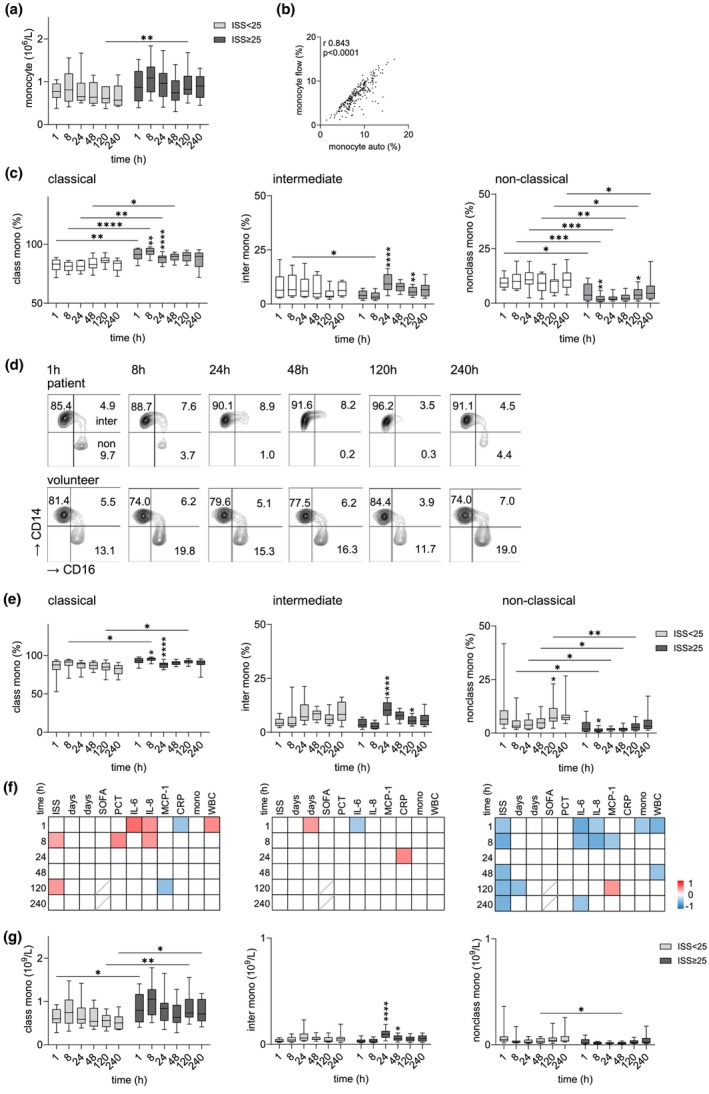
Circulating monocyte subsets in traumatised patients 1–240 h after injury. **(a)** Absolute numbers of circulating monocytes in patients with an ISS < 25 and ISS ≥ 25 determined by an automatic analyser. **(b)** Correlation of the percentage of monocytes within all leukocytes (set 100%) quantified by flow cytometry (flow) and an automatic analyser (auto); *n* = 43 patients, *n* = 243 data points, Spearman correlation coefficient *r* and *P* value are shown. **(c)** Time course of the percentage of monocyte subsets in 100% CD14^+^ monocytes in volunteers and all traumatised patients. **(d)** Time course of monocyte subsets in one typical patient (ISS 41) and one uninjured volunteer; flow cytometry, the percentage of positive cells in CD14^+^ monocytes is indicated in the respective quadrants. **(e)** Time course of the percentage of the monocyte subsets in both patient groups. **(f)** Correlation of the percentage of the monocyte subset in 100% CD14^+^ monocytes to clinical parameters in traumatised patients. In the correlation matrix, square colour indicates the magnitude of correlation; only significant correlations (Spearman) are shown. **(g)** Absolute numbers of circulating monocyte subsets in both patient groups. The percentage of the various monocyte subsets in 100% CD14^+^ monocytes, obtained by flow cytometry, was calculated to the absolute number of circulating monocytes for each patient. **(a, c, e, g)** Comparison between groups (between volunteers and all patients, or between both patient groups) and comparison between consecutive time points in one group (only significant changes related to the previous time point are shown): mixed‐effects model, *P < 0.05, **P < 0.01, ***P < 0.001, ****P < 0.0001.

The absolute number of circulating monocytes was higher in very severely injured patients compared to those with less severe injuries at 120 h post trauma, with a similar trend observed at other time points (Figure 1a). The percentage of monocytes among total leukocytes, as quantified by both an automated blood analyser and flow cytometry, showed a strong correlation (Figure 1b). This correlation validates the use of flow cytometry‐derived monocyte data in relation to absolute cell counts.

Typically, classical monocytes comprise 80–90% of total monocytes, while intermediate and non‐classical monocytes account for approximately 5–10% each.^16^ In uninjured volunteers, the proportions of these monocyte subsets remained stable within the CD14^+^ monocyte population over the 240‐h observation period (Figure 1c). Following trauma, the percentage of classical monocytes increased until 48 h post‐injury, whereas non‐classical monocytes remained markedly reduced throughout the entire post‐traumatic period. These changes are illustrated in Figure 1d, which shows monocyte subset dynamics in a representative trauma patient compared to an uninjured volunteer.

Comparing both patient groups, very severe injury induced a more pronounced phenotypic shift in monocytes than less severe injury (Figure 1e). The percentage of classical monocytes was significantly higher at 8 and 120 h post trauma, while non‐classical monocytes were nearly depleted, particularly between 8 and 120 h following very severe injury. Consequently, the percentage of non‐classical monocytes correlated inversely with injury severity score (ISS) at most time points and, in the early post‐injury phase, also with pro‐inflammatory cytokine levels (Figure 1f). These post‐traumatic changes in monocyte subsets were also reflected in their absolute cell numbers (Figure 1g).

### Marked post‐traumatic, injury severity‐dependent decrease in HLA‐DR expression on classical monocytes

**Figure 2:**
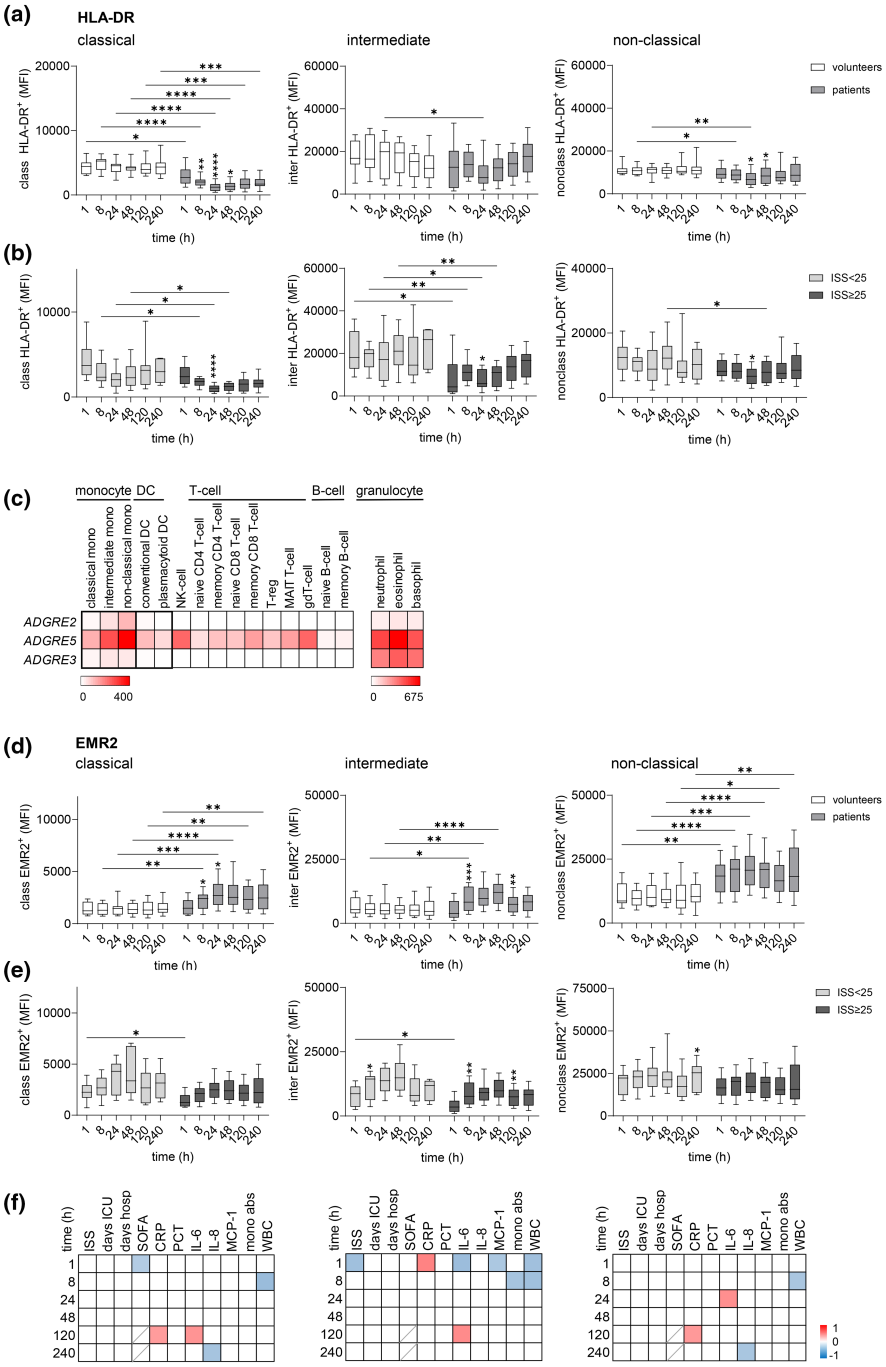
Expression of HLA‐DR and EMR2 at circulating monocyte subsets in traumatised patients 1–240 h after injury. **(a, b)** Expression of HLA‐DR (median fluorescence intensity, MFI) in classical, intermediate, and non‐classical monocytes in **(a)** uninjured volunteers and all traumatised patients, and in **(b)** patients with an ISS < 25 and ISS ≥ 25 1–240 h after trauma. **(c)** Expression of *ADGRE2*, *ADGRE5*, and *ADGRE3* in blood leukocyte subsets; RNA‐sequencing data Human Protein Atlas (proteinatlas.org)^20^, ^21^; mean from three different donors; transcripts per kilobase million (TPM) are shown. **(d, e)** Expression level of EMR2 (MFI) in classical, intermediate, and non‐classical monocytes of **(d)** uninjured volunteers and all traumatised patients, and of **(e)** patients with an ISS < 25 and ISS ≥ 25 1–240 h after trauma. **(a, b, d, e)** Comparison between groups and between consecutive time points in one group (only significant changes related to the previous time point are shown): mixed‐effect model, *P < 0.05, **P < 0.01, ***P < 0.001, ****P < 0.0001. **(f)** Correlation of the EMR2 levels in each monocyte subset (in 100% CD14^+^ monocytes) with clinical parameters after trauma. In the correlation matrix, square colour indicates the magnitude of correlation; only significant correlations (Spearman) are shown.

It is well known that trauma impairs the ability of monocytes to express HLA‐DR (MHC class II).^17^, ^18^, ^19^ Indeed, our patients exhibited much lower HLA‐DR levels compared to uninjured volunteers, particularly on classical monocytes (Figure 2a). This effect was injury‐dependent, as indicated by the lower HLA‐DR levels in monocytes of very severely injured patients compared to those with less severe injuries (Figure 2b).

### EMR2, CD97 and EMR3 levels increased in the sequence classical, intermediate to non‐classical monocytes in patients and volunteers

**Figure 3:**
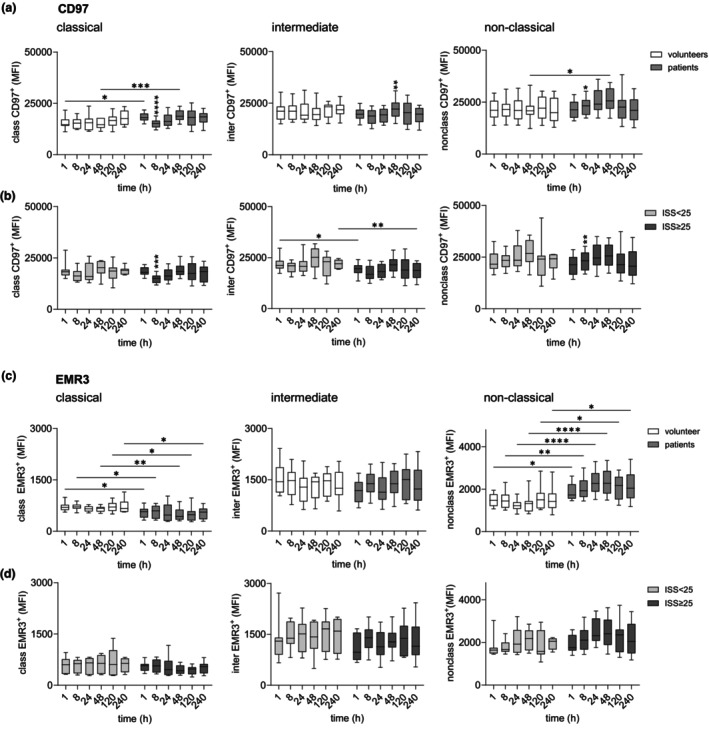
Expression of CD97 and EMR3 in circulating monocyte subsets in traumatised patients 1–240 h after injury. **(a, b)** Expression of CD97 (median fluorescence intensity, MFI) in classical, intermediate, and non‐classical monocytes in **(a)** uninjured volunteers and all traumatised patients, and in **(b)** patients with an ISS < 25 and ISS ≥ 25 1–240 h after trauma. **(c, d)** Expression of EMR3 (MFI) in the monocyte subsets of **(c)** uninjured volunteers and all traumatised patients, and of **(d)** patients with an ISS < 25 and ISS ≥ 25 1–240 h after trauma. **(a–d)** Comparison between groups and between consecutive time points in one group (only significant changes related to the previous time point are shown): mixed‐effect model, *P < 0.05, **P < 0.01, ***P < 0.001, ****P < 0.0001.

Next, we quantified EMR2, CD97 and EMR3 expression in monocyte subsets. Reanalysis of transcriptomic data revealed that all three adhesion GPCRs exhibited the lowest expression in classical monocytes and the highest in non‐classical (Figure 2c). However, expression levels varied among receptors: *ADGRE3* showed relatively low expression, *ADGRE2* was moderately expressed, and *ADGRE5* exhibited the highest expression. These findings were confirmed by flow cytometry. EMR2 expression increased nearly 5‐fold across monocyte subsets in both trauma patients and volunteers, following the sequence classical, intermediate, non‐classical (Figure 2). CD97 levels increased slightly in this order (Figure 3), with all monocyte subsets being strongly CD97‐positive^+^. EMR3 expression tripled from classical to non‐classical monocytes but remained consistently low (Figure 3).

### In all monocyte subsets, EMR2 levels increased, mainly independent of injury severity after trauma

In uninjured volunteers, EMR2, CD97 and EMR3 expression remained stable across monocyte subsets over a 240‐h period. Trauma induced significant alterations in EMR2 and EMR3 expression levels. EMR2 expression increased across all monocyte subsets, peaking at 24–48 h (Figure 2d). This course was observed in all patients and was largely independent of injury severity (Figure 2e and f). However, in very severely injured patients, the EMR2 increase was less pronounced, with lower levels detected immediately after injury in classical and intermediate monocytes. Consistently, post‐traumatic EMR2 expression in monocyte subsets showed no significant correlation with most clinical parameters, except for EMR2 levels on intermediate monocytes at 1 h post injury.

CD97 expression increased slightly post‐traumatically in both classical and non‐classical monocytes (Figure 3a). Patient groups hardly differ from one another (Figure 3b).

EMR3, which is expressed at much lower levels on monocytes than EMR2 and CD97, showed a sustained decrease on classical and, simultaneously, a marked prolonged increase on non‐classical monocytes throughout the entire post‐traumatic period (Figure 3c). No significant differences in EMR3 levels were observed between patient groups (Figure 3d). Consequently, monocytic CD97 and EMR3 levels did not correlate with clinical parameters (not shown).

### The posttraumatic decrease of circulating DCs correlates with adverse clinical parameters late after injury

Circulating DCs are rare, comprising < 1% of total leukocytes. In this study, we quantified the proportion of cDCs (CD11c^+^ CD123^−^) and pDCs (CD11c^−^ CD123^+^) among CD45^+^ leukocytes and assessed EMR2 and CD97 expression on these subsets. Notably, DCs lack EMR3.

**Figure 4:**
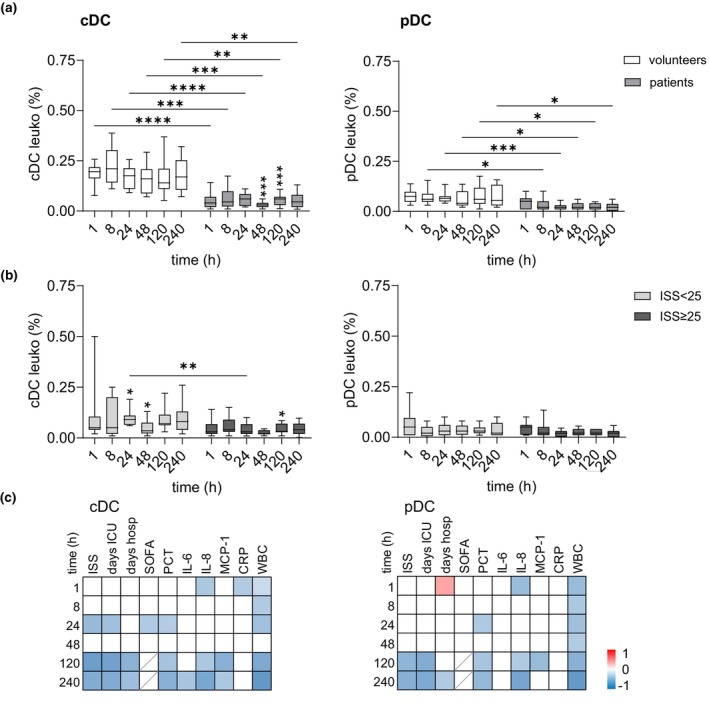
Circulating DC subsets in traumatised patients 1–240 h after injury. **(a, b)** Time course of the percentage of CD11c^+^ CD123^−^ cDCs and CD11c^−^ CD123^+^ pDCs among CD45^+^ leukocytes (set at 100%) in **(a)** volunteers and all patients, and **(b)** in both patient groups after traumatic injury. Comparison between groups and between consecutive time points in one group (only significant changes related to the previous time point are shown): mixed‐effect model, *P < 0.05, **P < 0.01, ***P < 0.001, ****P < 0.0001. **(c)** Correlation of the percentage of cDCs and pDCs to clinical parameters. In the correlation matrix, square colour indicates the magnitude of correlation; only significant correlations (Spearman) are shown.

The percentage of cDCs and pDCs was obviously reduced after injury compared to uninjured volunteers (Figure 4a). Very severe injury tended to cause a more pronounced loss of cDCs, particularly beyond 24 h post trauma (Figure 4b). The percentage of both DC subsets was inversely correlated with white blood cell (WBC) counts at nearly all time points (Figure 4c). The decline in DC percentages correlated with higher ISS, prolonged intensive care unit (ICU) and hospital stays, and elevated pro‐inflammatory cytokine and procalcitonin (PCT) levels, particularly at later time points 120–240 h post injury.

**Figure 5:**
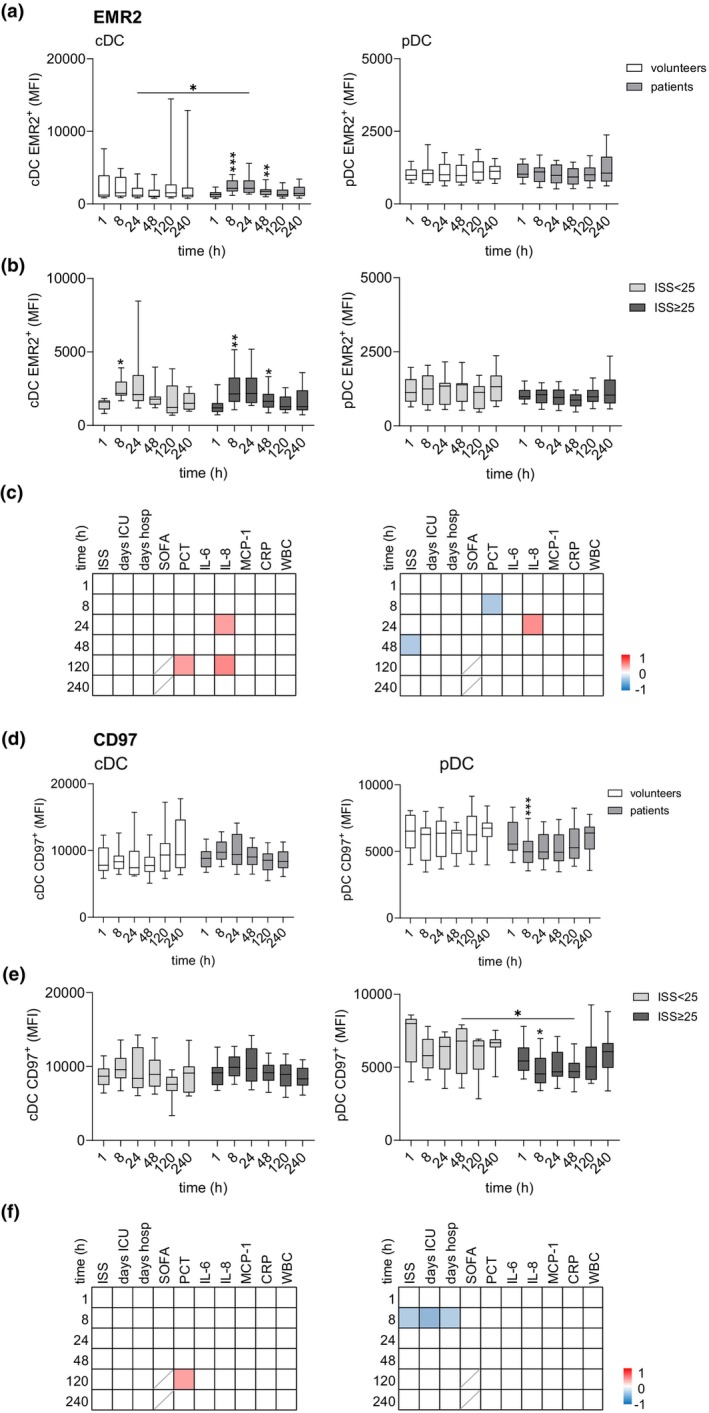
Expression of EMR2 and CD97 in circulating DC subsets of traumatised patients 1–240 h after injury. **(a, b)** Expression of EMR2 (median fluorescence intensity, MFI) in cDCs and pDCs in volunteers and all patients **(a)** and in the two patient groups **(b)** 1–240 h after injury. **(c)** Correlation of the expression level of EMR2 in cDCs and pDCs to clinical parameters. In the correlation matrix, square colour indicates the magnitude of correlation; only significant correlations (Spearman) are shown. **(d, e)** Expression of CD97 (MFI) in cDCs and pDCs in volunteers and all patients **(d)** and in the two patient groups **(e)** 1–240 h after injury. **(f)** Correlation of the expression level of CD97 in cDCs and pDCs to clinical parameters (refer **c**). **(a, b, d, e)** Comparison between groups and between consecutive time points in one group (only significant changes related to the previous time point are shown): mixed‐effect model, *P < 0.05, **P < 0.01, ***P < 0.001.

In both volunteers and trauma patients, circulating DCs exhibited moderate EMR2 and high CD97 expression (Figure 5). While volunteers maintained stable EMR2 and CD97 expression in DCs over the 240‐h period, substantial inter‐individual variability was observed. In trauma patients, regardless of ISS, EMR2 expression on cDCs slightly increased at 8 h post injury, remained elevated until 24 h, and then declined (Figure 5a and b). EMR2 expression on pDCs remained unchanged. EMR2 levels in both DC subsets did not correlate with clinical parameters (Figure 5c). CD97 expression remained unchanged in cDCs after trauma (Figure 5d). In pDCs, CD97 changed slightly post‐injury; at 8 h, CD97 levels correlated inversely with ISS, as well as with ICU and hospital length of stay (Figure 5f). Overall, the changes in EMR2 and CD97 levels on DCs were rather small after trauma.

## Discussion

DCs constitute approximately 1% of peripheral blood mononuclear cells.^22^ Here, we show that cDCs and pDCs nearly vanished from the circulation of trauma patients, especially in those with higher ISS. At 120 and 240 h post injury, DC levels inversely correlated with ICU and hospital length of stay and inflammation markers, suggesting DC loss relates to poor outcomes. Where do DCs go? A recent mouse study provides valuable insights.^23^ Single‐cell RNA sequencing was performed on brain‐derived microglia and infiltrating circulating leukocytes 4 days after traumatic brain injury. The data show increased infiltration of circulating DCs and monocytes/macrophages in the brain, whereas T, NK, and B cell populations remained unchanged. Another study demonstrates that trauma alters DC distribution and induces imbalances among DC subsets in both lymphoid and non‐lymphoid organs.^24^ These findings indicate that DCs migrate into tissues post‐trauma. EMR2 and CD97 expression on DCs changed only slightly with trauma. Interestingly, nanoparticles, likely binding CD97 on mouse bone marrow‐derived DCs, activated mechano‐sensitive K+ channels and the formation of the NLRP3 inflammasome,^25^ which serves as an intracellular DAMP sensor in innate immune cells.^26^

Monocytes constitute 2–8% of circulating leukocytes. As shown here and previously reported,^27^ very severe injury led to an increase in circulating monocytes, though much less pronounced than the dramatic rise in neutrophils.^9^ CD14^+^ monocytes initially shifted toward the classical subset, known for their superior phagocytic capacity in clearing necrotic debris,^28^ a function crucial following injury. Classical monocytes are the first to be released from the bone marrow, which explains their immediate surge after trauma. Contrary to our findings, a recent study on trauma patients reported that within the first 12 h after injury, approximately 50% of all monocytes were CD14^hi^ CD16^+^, classifying them as intermediate monocytes.^29^ In uninjured volunteers, the same study observed around 30% intermediate monocytes. These proportions differ markedly from both previously published data on healthy individuals^16^, ^28^, ^30^ and our data. The discrepancy is likely because of a brief *in vitro* lipopolysaccharide pre‐stimulation of monocytes in the cited study.^29^

Each examined adhesion GPCR, though expressed at different levels, shows the highest expression in non‐classical monocytes in both volunteers and patients. Genes overexpressed in these monocytes are involved in cytoskeletal dynamics, motility, and infiltration^4^, ^31^ supporting their roles in vessel patrolling and tissue infiltration. The upregulation of EMR2 and EMR3 on non‐classical monocytes likely facilitates adhesion to injured surfaces and extravasation after trauma. EMR2 transiently increased in an injury‐independent manner across all monocyte subsets 24–48 h after injury, following a time course similar to that observed in neutrophils^9^ and cDCs. The uniform posttraumatic upregulation of EMR2 across most innate immune cells suggests a shared regulatory mechanism, potentially driven by DAMPs. Both *in vivo* and *in vitro* data from primary monocytes and neutrophils^9^, ^10^, ^32^ indicate that DAMPs induce EMR2 upregulation.

Paradoxically, trauma leads to adverse functional changes in monocytes. Injury severity affects their ability to express HLA‐DR, which is crucial for presenting antigens to naïve CD4^+^ T cells.^17^, ^18^, ^19^, ^33^ This injury‐dependent decline in HLA‐DR was also observed in our trauma patients. It is likely that the less pronounced increase of EMR2 in very severely injured compared to less injured patients is also associated with unfavorable functional changes in the monocytes after injury. Notably, persistently low HLA‐DR levels are linked to a higher risk of sepsis, whereas patients with uncomplicated recovery show normalisation post‐trauma.^18^ Moreover, surface expression of TLR2, TLR4, and TLR9, receptors for DAMPs and pathogen‐associated molecular patterns (PAMPs), was impaired in monocytes from severely injured patients.^33^ Trauma also reduces cytokine production and phagocytic activity. Lipopolysaccharide‐stimulated CD14^+^ monocytes from trauma patients released lower TNF‐α and IL‐1β levels, correlating with reduced *NLRP3* expression.^19^, ^34^, ^35^ Phagocytic capacity was diminished shortly after hospital admission and 1 day post injury.^36^ These findings were confirmed in a porcine polytrauma model, where monocytes showed reduced TLR2 expression, impaired maturation and decreased bacterial clearance.^37^ Overall, these trauma‐induced impairments of monocytic innate immunity increase susceptibility to infections and/or multiorgan dysfunction.^38^

CD97 was only slightly regulated on blood DC and monocyte subsets after trauma. What is known about CD97 on circulating immune cells in clinical settings? To evaluate biomarkers distinguishing patients with sepsis from those with non‐infectious systemic inflammatory response syndrome (SIRS), CD97 and EMR2 expression on neutrophils were quantified within 72 h of ICU admission.^13^ CD97 levels were similar between patients and healthy subjects, regardless of sepsis status. In contrast, neutrophils from sepsis patients showed a higher prevalence of EMR2 expression compared to those from non‐septic patients. However, another study reported elevated CD97 expression on neutrophils in septic patients during days 1–3 after hospital admission, compared to burn trauma patients and age‐matched uninjured volunteers.^39^ Furthermore, *ADGRE5* expression was higher in circulating neutrophils of patients with bloodstream infections caused by *Staphylococcus aureus* compared to healthy subjects.^40^ These partly contradictory findings are likely because of variations in blood sampling time points, differences in patient cohorts, and the diversity of pathogens responsible for sepsis.

Overall, EMR3 has only been studied *in vitro* in leukocytes^14^, ^15^; clinical studies are lacking. Compared to its closest relatives, EMR3 levels are relatively low across all monocyte subsets but increase in the sequence from classical to non‐classical monocytes. On the one hand, after trauma, EMR3 expression decreased in classical monocytes. In contrast to CD97 and EMR2, CD34^+^ CD33^+^/CD38^+^ progenitors in the bone marrow and CD34^+^ CD33^−^/CD38^−^ committed haematopoietic stem cells do not express EMR3.^15^ Likely, the early post‐traumatic decrease of EMR3 on classical monocytes is a sign of the increased monocyte release from the bone marrow and/or generation of monocytes from progenitors. EMR3 is a marker of mature granulocytes.^15^ On the other hand, there is a marked upregulation of EMR3 on non‐classical monocytes in all injured patients, similarly to the upregulation seen for EMR2 across all monocyte subsets. This likely indicates that EMR3 is also upregulated by DAMPS and is used for migration out of circulation.

### Conclusions, relevance to diagnosis, treatment and management

The presence and composition of blood DCs and monocyte subsets change significantly after trauma. Here, we demonstrate for the first time that DCs nearly disappear from circulation, a phenomenon associated with adverse clinical parameters late in the post‐traumatic course. Studies with follow‐up beyond 10 days are needed to assess whether blood DC counts can serve as a prognostic marker for patient recovery and outcomes after severe injury.

The adhesion GPCRs EMR2 and EMR3 are involved in the innate immune response to injury and are likely regulated by DAMPs. EMR2 is strongly upregulated on neutrophils^9^ and all monocyte subsets, while EMR3 is markedly upregulated on non‐classical monocytes after trauma. Identifying the DAMPs involved, elucidating the signalling pathways, and clarifying the activation mechanism of EMR3, since that of EMR2 is already well understood, will enhance our knowledge of innate immune responses post‐injury and offer opportunities for monitoring and intervention.

### Limitations of the study

The sample size of this study is relatively small. Nonetheless, trauma remains an attractive model for studying innate immune responses, as it typically affects otherwise healthy individuals and has a well‐defined onset. DAMP clearance follows a consistent, time‐matched pattern across injured patients. We showed that both monocytic and neutrophilic^9^ EMR2 are consistently upregulated after trauma once a certain injury threshold is reached.

The study included patients with varying injury severity scores. While this allows analysis across a broad range, it may also introduce variability. However, we confirmed well‐established injury‐related patterns, such as increased monocyte numbers and HLA‐DR downregulation on monocytes. Therefore, our cohort is appropriate for identifying clinically relevant changes in leukocyte subsets and adhesion GPCR expression.

## Methods

### Ethics statement and clinical study

This prospective study was approved by the local ethics committee at Leipzig University (reference numbers 188‐17lk) and conducted in accordance with the Declaration of Helsinki. Samples and data were collected with informed written consent from patients or their legal representatives, as well as from uninjured volunteers.

Traumatic injury refers to sudden and severe physical injuries requiring immediate medical attention. Patients were excluded from the study if they met any of the following criteria: age < 18 years, time from trauma to hospital admission > 1 h, life expectancy < 24 h, participation in other clinical trials, cardiopulmonary resuscitation at the accident scene, immediate death after hospital admission, known or suspected pregnancy, or a history of radio‐ or chemotherapy within the last 3 months.

The ISS was used to define trauma and assess its severity.^41^ In our study, the ISS, initially estimated based on whole‐body computed tomography and confirmed retrospectively after hospital discharge, ranged from 9 to 66, categorising patients from moderately to very severely injured.^42^ Patients were divided into two groups: ISS < 25 (9–24) and ISS ≥ 25. The SOFA score, predicting outcome in critically ill patients,^43^ could be calculated up to 48 h after injury for all patients, as those with a low ISS, in particular, were discharged from the ICU early.

CD97 expression was analysed in the same 34 trauma patients previously investigated for EMR2^9^ (Table 1). For EMR3, we included 13 of these 34 patients (13/34) and an additional 10 trauma patients (*n* = 23). The ISS < 25 and ISS ≥ 25 groups had comparable characteristics (Table 1). Additionally, the study included uninjured volunteers (*n* = 10; median age: 56.0 years [42.5–64.0]; 3/10 female).

**Table 1:** Characteristics of the traumatised patients

	EMR2, CD97			EMR3		
	ISS < 25	ISS ≥ 25	*P* value*	ISS < 25	ISS ≥ 25	*P* value*
Patients (*n*)	9	25		8	15	
ISS	17 (10.5–18.0)	38 (30.5–44.0)	< 0.0001	15.5 (9.5–20.8)	34.0 (29.0–41.0)	< 0.0001
Age (years)	65.0 (39.0–74.0)	53.0 (32.5–68.0)	0.381	65.5 (44.5–80.0)	40.0 (34.0–56.0)	0.055
Sex, female (*n*)	2	8	0.692**	1	4	0.621**
Days at ICU (*n*)	2.0 (1.5–3.5)	11.0 (5.0–15.5)	0.0001	1.5 (0–3.8)	7.0 (4.0–17.0)	0.002
Days in hospital (*n*)	15.0 (11.0–28.0)	19.0 (13.0–35.5)	0.339	11.0 (6.3–19.5)	23.0 (10.0–41.0)	0.077

ICU, intensive care unit; ISS, injury severity score.

* Mann–Whitney *U*‐test.

** Fisher's exact test.

### Blood sample preparation and determination of parameters

Blood samples from patients were collected at 1, 8, 24, 48, 120 and 240 h after hospital admission, with a tolerance of ±10% for each time point. Uninjured volunteers underwent the same blood sampling schedule as the patients.

Leukocytes were isolated within 15 min of blood draw. A 2.5‐mL sample of EDTA‐treated blood was mixed with 22.5 mL of red blood cell lysis solution^44^ and incubated for 10 min at 4°C. The cells were then washed twice with PBS (pH 7.4) by centrifugation at 300× *g*. In trauma patients, white blood cell (WBC) counts were measured in EDTA‐treated blood at each time point using an automated blood cell analyser (XN‐9000, Sysmex GmbH, Norderstedt, Germany).

To obtain serum or EDTA plasma, the respective Monovette (Sarstedt AG, Nümbrecht, Germany) was centrifuged at 2000× *g* for 10 min at 20°C. The supernatants were transferred into encoded cryotubes, stored at −80°C, and thawed only once for analysis. IL‐6, IL‐8 and MCP‐1 were quantified in sera using the human IL‐6, IL‐8, MCP‐1 BD OptEIA ELISA kits (BD Biosciences, Heidelberg, Germany). C‐reactive protein (CRP) was quantified in sera by immunoturbidimetry, and procalcitonin (PCT) in plasma by the TRACE technology (Roche Deutschland Holding GmbH, Grenzach‐Wyhlen, Germany).

### Flow cytometric analysis of patients leukocytes

The antibodies (Abs) used in this study are summarised in Table 2. Each Ab was titrated to determine the optimal concentration for establishing multi‐colour Ab panels targeting circulating myeloid cells, with a focus on monocytes and DCs. The concentration of each Ab is 0.05–0.2 μg per 1 × 10^6^ cells in 100 μL staining volume. The staining procedure, controls, and measurement have been previously described.^9^ Briefly, at each time point, two samples of 1.4 × 10^6^ leukocytes were prepared. The cells were first blocked to prevent Fc‐binding and then stained with the Ab panel for 30 min at 4°C. The first sample contained all Abs, while in the second sample, adhesion GPCR Abs were replaced by isotype‐specific control Abs labelled with the corresponding fluorophores. Cells were washed three times, filtered through a 70 μm strainer, and immediately analysed without fixation using a BD LSRFortessa™ X‐20 Cell Analyzer with BD FACSDiva™ software (BD Biosciences). To minimise batch effects across different days, the flow cytometer was calibrated before each run using Sphero™ Rainbow Calibration Particles (BD). The number of analysed cells was determined based on the counted pDCs within the respective gate (CD11c^−^ CD123^+^), with a minimum of 1 × 10^3^ cells required. The minimum measurement time was 6 min at a low flow rate.

**Table 2:** Antibodies (Abs) and dyes used in flow cytometry

Antigen, fluorophore	Clone	Company, catalogue no.
CD11c PE‐Cy7	B‐Ly6	Becton Dickinson GmbH (BD, Heidelberg, Germany), 561 356
CD14 BUV395	MφP9	BD, 563561
CD16 BUV737	3G8	BD, 612786
CD3 BV510^a^	UCHT1	BD, 563109
CD19 BV510^a^	SJ25C1	BD, 562947
CD20 BV510^a^	2H7	BD, 563067
CD45 BB515	HI30	BD, 564585
CD97 BV421	VIM3b	BD, 742445
CD123 BV605	7G3	BD, 564197
EMR2 Alexa‐Fluor‐647^b^	2A1	Bio‐Rad Lab GmbH (Feldkirchen, Germany), MCA2330A647T
EMR3 Alexa‐Fluor‐647^b^	3D7	Bio‐Rad, MCA2476A647
HLA‐DR PerCP‐Cy5‐5	G46‐6	BD, 560652

^a^ BV510 dump channel: CD3, CD19 and CD20 identify T and B cells, which are not of interest in this study; only CD45^+^ CD3/19/20^−^ cells are carried forward for analysis.

^b^ EMR2 and EMR3 were measured, always together with CD97, in two blood samples.

The datasets from all individuals were analysed using FlowJo Software 10 (FlowJo LLC, Ashland, OR, USA). To ensure fluorescence measurement consistency over extended periods, the FlowClean plugin was applied, automatically detecting and removing abnormal events based on the time channel, thereby enhancing data stability. Multiple threshold‐based quality control techniques were employed. Sample quality was assessed using the Sample Quality Check function in FlowJo. Doublet discrimination was performed using FSC‐H vs. FSC‐A gating. FSC and SSC thresholds were set to remove electronic noise, and a minimum FSC threshold was applied to exclude debris. Finally, SSC/FSC scatter analysis was used to refine the dataset to single, viable cells.

**Figure 6:**
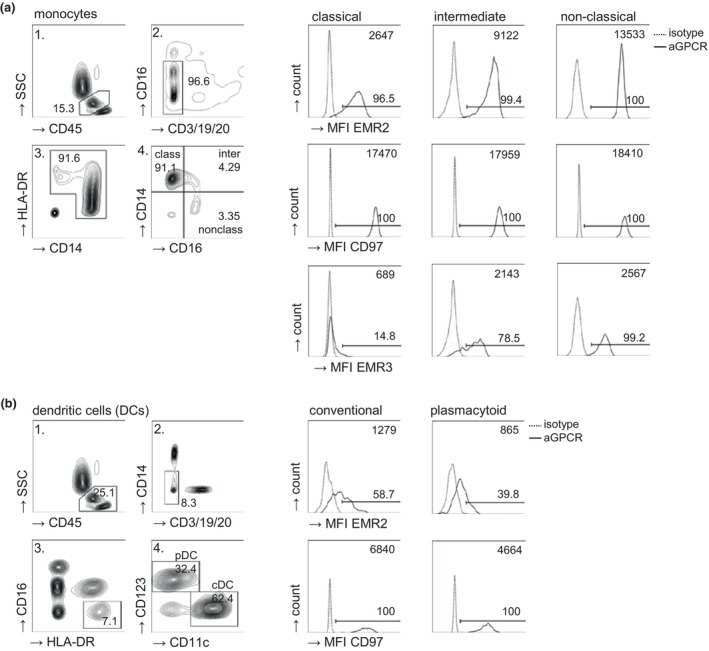
Characterisation of circulating monocyte and DC subsets for EMR2, CD97, and EMR3 expression in flow cytometry. **(a, b)** Analysis of EMR2, EMR3, and CD97 in **(a)** monocytes (CD14^hi^ CD16^−^ classical, CD14^hi^ CD16^+^ intermediate, CD14^lo^ CD16^+^ non‐classical) and **(b)** DC subsets (CD11c^+^ CD123^−^ conventional, CD11c^−^ CD123^+^ plasmacytoid); DCs are EMR3‐negative. Left: The main gating strategy is shown in single steps. The percentage of cells within a figure is set to 100%, and the percentage of gated cells, further examined, is indicated. Right: Median fluorescence intensity (MFI; right‐upper corner) of isotype control‐on and adhesion GPCR antibody‐stained cells; the percentages of positive cells are indicated at the gate. Exemplary data of an injured patient (ISS 32) 24 h after trauma are shown.

The primary gating and analysis strategy for circulating monocytes and DCs is illustrated in Figure 6. Leukocyte subsets were distinguished in each sample primarily through scatter analysis and staining with CD45, CD14, CD16, HLA‐DR, and CD3/CD19/CD20 Abs. Monocytes were classified into classical (CD14^hi^ CD16^−^), intermediate (CD14^hi^ CD16^+^) and non‐classical monocytes (CD14^lo^ CD16^+^), collectively accounting for 100% of CD14^+^ monocytes. Blood DCs (CD45^+^ HLA‐DR^+^ CD14^−/lo^ CD16^−^ CD3/CD19/CD20^−^) were further separated into cDC and pDC using CD11c/CD123 staining, and their proportions were quantified relative to total CD45^+^ leukocytes, set at 100%. Each subset was analysed for the percentage of adhesion GPCR^+^ cells and/or the median fluorescence intensity (MFI) of adhesion GPCR expression. A representative analysis for EMR2, CD97, and EMR3 is shown in Figure 6.

### Statistics

Data were analysed using SPSS v27 (IBM, Armonk, NY) and GraphPad Prism 10.2 (GraphPad Software, Boston, MA). Normally distributed continuous variables were expressed as mean ± standard error of the mean (SEM), while non‐normally distributed variables were presented as median with interquartile range (25th–75th percentile). Box plots are shown as mean/25–75% percentiles with 10–90 percentile whiskers. If no patient sample was available at a certain time point because the patient underwent surgery or other medical treatments or had already left the hospital, this sample was treated as missing. Thus, a mixed‐effect model approach was used. We corrected for multiple comparisons by controlling the False Discovery Rate using the two‐stage step‐up method of Benjamini, Krieger, and Yekutieli. All *P* values were two‐sided, and statistical significance was set at *α* < 0.05.

## Author contributions

**Leyu Zheng:** Formal analysis; investigation. **Carolin Fuchs:** Resources. **Christian Kleber:** Resources. **Georg Osterhoff:** Conceptualization; resources. **Gabriela Aust:** Conceptualization; formal analysis; methodology; resources; supervision; writing – original draft.

## Conflict of interest

The authors declare no conflict of interest.

## Data Availability

The original data are available upon request from the authors. GA had full access to all the data in the study and takes responsibility for the integrity of the data and the accuracy of the data analysis.
